# Fabry Cardiomyopathy: Current Treatment and Future Options

**DOI:** 10.3390/jcm10143026

**Published:** 2021-07-07

**Authors:** Irfan Vardarli, Manuel Weber, Christoph Rischpler, Dagmar Führer, Ken Herrmann, Frank Weidemann

**Affiliations:** 1 Department of Medicine I, Klinikum Vest GmbH, Knappschaftskrankenhaus Recklinghausen, Academic Teaching Hospital, Ruhr-University Bochum, 45657 Recklinghausen, Germany; frank.weidemann@klinikum-vest.de; 2 Department of Nuclear Medicine, University Hospital Essen, 45147 Essen, Germany; manuel.weber@uk-essen.de (M.W.); christoph.rischpler@uk-essen.de (C.R.); ken.herrmann@uk-essen.de (K.H.); 3 Department of Endocrinology, Diabetes and Metabolism, Clinical Chemistry—Division of Laboratory Research, Endocrine Tumor Center, WTZ/Comprehensive Cancer Center, University Hospital Essen, University of Duisburg-Essen, 45147 Essen, Germany; dagmar.fuehrer@uk-essen.de

**Keywords:** Fabry, cardiomyopathy, treatment, options

## Abstract

Fabry disease is a multisystem X-linked lysosomal storage disorder caused by a mutation in the alpha-galactosidase A gene. Deficiency or reduced activity of alpha-galactosidase A (GLA) is leading to progressive intracellular accumulation of globotriaosylceramide (GL3) in various organs, including the heart, kidney and nerve system. Cardiac involvement is frequent and is evident as concentric left ventricular hypertrophy. Currently, the standard treatment is enzyme replacement therapy or chaperone therapy. However, early starting of therapy, before myocardial fibrosis has developed, is essential for long-term improvement of myocardial function. For future treatment options, various therapeutic approaches including gene therapy are under development. This review describes the current and potential future therapy options for Fabry cardiomyopathy.

## 1. Introduction

Fabry disease (FD) is a multisystem X-linked lysosomal storage disorder caused by a mutation in the alpha-galactosidase A (GLA) gene [1]. Deficiency or reduced activity of GLA is leading to progressive accumulation of intracellular globotriaosylceramide (GL3) in various organs, including the heart, kidney and nerve system [2]. Typical manifestations include neuropathic pain, telangiectasias, anhidrosis, gastrointestinal symptoms, cornea verticillata, renal failure with unknown etiology, unexplained left ventricular (LV) hypertrophy or neurological manifestations (e.g., cryptogenic stroke) [3,4,5,6,7,8]. Regarding diagnosis of FD and Fabry cardiomyopathy, various reviews have been published [9,10].

In suspected cases, determination of GLA activity is recommended. In males, GLA activity <1% is highly suggestive for the disease of classic FD [9]. In females and in patients with late-onset mutations (e.g., N215S cardiac variant mutation) the enzyme activity may be residual or even normal; thus, in such cases, genetic testing of Fabry mutations is mandatory [11]. Basically, the additional determination of globotriaosylsphingosine (lyso-GL3) is recommended. Lyso-GL3 levels ≥2.7 ng/mL are associated with classical mutations [12]. For evaluation of relevant cardiac involvement, the determination of highly sensitive Troponin (hsTnT) and B-type natriuretic peptide (NT proBNP), as biomarkers, are useful [13]. In FD, hsTNT is more related to early development of cardiac fibrosis and NT proBNP to heart failure in advanced stages [14].

Although blood tests are very easy to perform, a lot of patients are diagnosed late during the disease progression. The reason for this is that symptoms can vary a lot and thus it is difficult for the clinician to assign very general symptoms to this very rare disease. Thus, overall it takes on average 10 years from the first symptom to the correct diagnosis of FD.

In patients with FD, morbidity and poor prognosis are mainly driven by cardiomyopathy [15,16]. Currently, the standard treatment is enzyme replacement therapy or chaperone therapy [17]. However, early beginning of therapy, before myocardial fibrosis has developed, is essential for long-term improvement or stabilization of myocardial function [18]. For future treatment options, various therapeutic approaches including gene therapy are under development. This review describes the current and future therapy options for Fabry cardiomyopathy.

## 2. Fabry Cardiomyopathy

Left ventricular (LV) hypertrophy is the main mechanism of the Fabry cardiomyopathy. LV hypertrophy (LVH) is partly a reaction of the tissue to the GL3 deposition [10]. In addition, an increase of trophic factors, e.g., lyso-Gb3, play a role in the development of Fabry cardiomyopathy [19,20,21,22,23,24,25,26]. Furthermore, upregulation of cellular adhesion molecules in vascular endothelial cells or by oxidative stress leads to LVH [27]. Genetic aspects should also be considered. Germain et al. confirmed that p.N215S is a disease-causing Fabry mutation with severe clinical manifestations essentially limited to the heart until late adulthood, especially in males [28].

**Figure 1:**
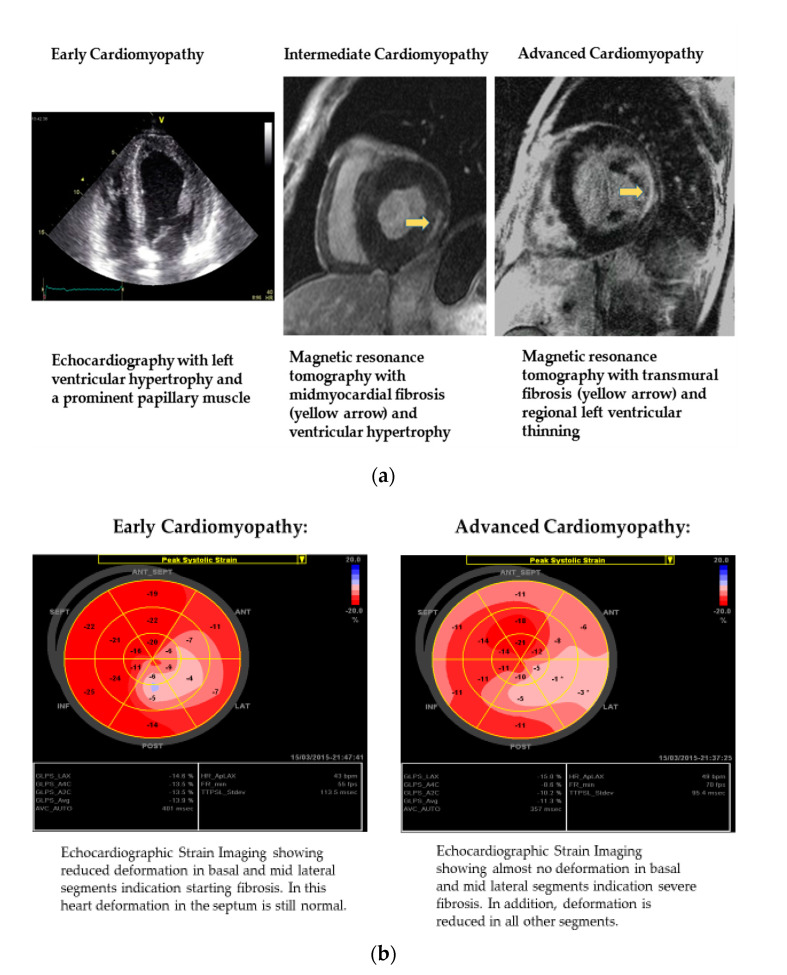
(**a**) Echocardiographic aspects and magnetic resonance tomography findings in various stages of Fabry cardiomyopathy. (**b**) Strain analysis in various stages of Fabry cardiomyopathy.

Concentric LV thickening without outflow tract obstruction [29,30,31], prominence of papillary muscles [30,32,33], preservation of ejection fraction and slight to mild to moderate impairment of diastolic function [15] are typical echocardiographic aspects in Fabry patients. Even in the early stages of FD, mild impairment of regional myocardial function is common, and can be assessed by strain rate or deformation imaging [16,19,29,34]. Typically, the reduction of myocardial longitudinal function starts in basal regions of posterolateral myocardium and is related to myocardial fibrosis in later stages [16]. In the end-stage cardiomyopathy, wall motion abnormalities can be observed on bidimensional echocardiography [35]. The development of replacement fibrosis, which is mostly limited to posterolateral segments of basal myocardium, is a typical morphologic sign for Fabry cardiomyopathy [19,22] and is associated with a poor prognosis [16,35]. This fibrosis can be assessed by gadolinium-contrast late enhancement (LE) magnetic resonance imaging (the gold standard for the assessment of myocardial fibrosis) or indirectly by functional deformation imaging [19,22,36,37]. Echocardiographic aspects and magnetic resonance tomography findings in various stages of Fabry cardiomyopathy were shown in Figure 1. In general, there are sufficient cardiac tools to assess early Fabry cardiomyopathy. In this context cardiac magnetic resonance tomography with T1 mapping and late enhancement imaging are very important imaging tools. Only in some patients with inconsistent diagnostic results and insufficient treatment effects cardiac biopsy might help.

Common misdiagnoses involving the heart and in particular the finding of LVH in FD are hypertensive heart disease, sarcomeric hypertrophic cardiomyopathy, cardiac amyloidosis and Friedreich cardiomyopathy. Whenever FD is suspected in hypertrophic hearts, additional questions about typical Fabry symptoms should be discussed with the patient. If the patient suffered from LVH and typical symptoms, Fabry disease is very likely and a blood test should be performed.

In case of unexplained LVH (>13 mm), FD should be suspected. In general male patients can develop LVH at the age of 20 and female patients around 10 years later. In a cohort of 100 males with unexplained LVH (≥13 mm) older than 30 years, Palecek et al. found a prevalence of 4.0% for FD. They recommend screening for FD in all men older than 30 years with unexplained LVH even in the absence of obvious extracardiac manifestations [38]. Hagège et al. investigated a cohort of 392 adult patients (278 men) with HCM defined by wall thickness ≥ 15 mm in 29 French cardiology centers. In four men (all older than 40 years; 1.5% of the cohort) the diagnosis of FD was confirmed by blood and genetic testing [39]. Nakao et al. and Sachdev et al. reported in their trials similar results [40,41]. However, other multicenter screening trials found lower prevalence of FD in patients with unexplained LVH, ranging between 0.5% and 1.5% [42,43].

In all patients with a Fabry cardiomyopathy regular follow-up examinations are necessary. For cardiac test echocardiography, ECG, Holter ECG, ergometry and cardiac biomarkers should be performed. If patients are treated, all these diagnostic tests should be performed once a year. An MRI including late enhancement imaging and if possible T1 mapping should be done at least every two years [44,45].

### 2.1. Treatment of Fabry Cardiomyopathy

#### 2.1.1. Current Treatment

**Figure 2:**
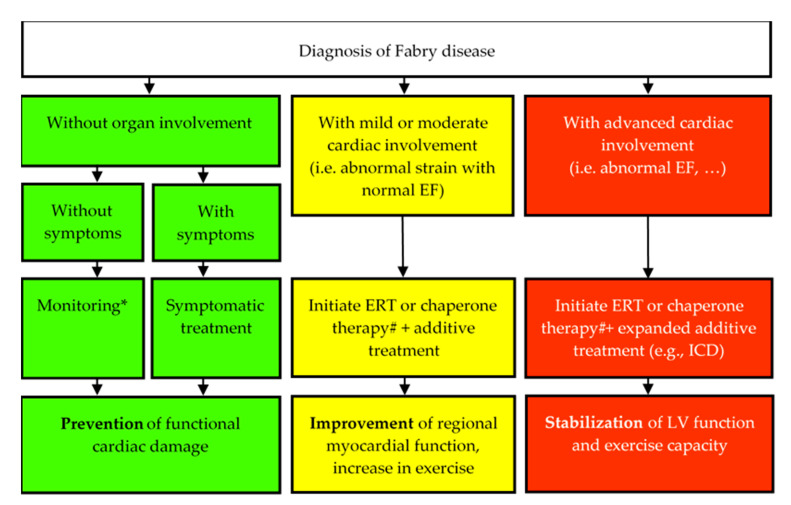
Current treatment algorithm for Fabry cardiomyopathy (adapted with permission from Weidemann F. et al. The Fabry cardiomyopathy: models for the cardiologist. Annu Rev Med 2011; 62: 59-67. Copyright^©^ 2021, Marketplace^TM^. #: only in patients with Migalastat-amenable GLA mutations possible. EF: ejection fraction, ERT: Enzyme replacement therapy, ICD: implanted cardioverter defibrillator, LV: left ventricular. * e.g., echocardiography, Holter electrocardiography (ECG). All established diagnostic tests should be performed one a year.

Fabry cardiomyopathy, which leads to reduced life expectancy in untreated patients [46,47], can be treated with a causal therapy. The current therapy for FD (Figure 2) is enzyme replacement therapy (ERT) or in amenable mutations Chaperone therapy. ERT is administered by lifelong biweekly infusion of recombinant enzyme [48,49], which is available as agalsidase alfa (Replagal^®^, Shire Human Genetics Therapies AB, Stockholm, Sweden, since 2019 Takeda Pharma AG, 8152 Opfikon, Switzerland) and agalsidase beta (Fabrazyme^®^, Sanofi Genzyme, Cambridge, MA, USA). After enzyme replacement, microvascular GL3 depositions were cleared in the kidneys, skin and the heart of most Fabry patients [49,50,51,52]. The depletion of GL3 depositions and reduced inflammation are associated with reduced LV mass and in some patients with early stage of the disease augmentation of regional myocardial function is possible [19,29,53]. Nevertheless, the effect of ERT on life expectancy remains unclear. The timing of ERT is important, as the foremost benefit of ERT was reported in patients with less severe stage of cardiomyopathy at baseline [18,20]. Improvements of cardiomyopathy (decrease in myocardial mass, improvement of regional myocardial function and increase in exercise capacity) were observed during three years of ERT only in patients without replacement fibrosis. A reduction of LVH and stabilization of exercise capacity and LV function were found in patients with myocardial fibrosis only in one LV segment; whereas no benefit in LV function and no clear regression of LVH were seen in patients with more than one affected (myocardial fibrosis) LV segment [19]. Most patients, particularly male patients, are developing antibodies with neutralizing activity during long-term ERT [54,55,56]. The impact of these neutralizing antibodies on heart morphology and function remains unclear.

Chaperone therapy with Migalastat (Galafold^®^, Amicus Therapeutics, Cranbury, NJ, USA) with its convenient oral regimen was approved in Europe in 2016, and is an important treatment option for FD in patients with migalastat-amenable GLA mutations [17]. Germain et al. showed significant decrease in the LV mass index after ≤24 months’ migalastat therapy [57]. However, as myocardial fibrosis is probably not reversible, starting treatment in early stage of the disease is crucial for improvement of prognosis [19]. Prescribing information and/or the migalastat amenability table at the website (https://www.galafoldamenabilitytable.com/hcp, accessed on 10 June 2021) should be considered for a list of amenable and non-amenable GLA mutations to migalastat. Migalastat is not recommended in patients with eGFR < 30 mL/min/1.73 m^2^ [58,59,60].

In all patients with a cardiomyopathy additional therapy is necessary. Optimization of hypertension treatment and control of proteinuria is required. In such cases angiotensin-converting enzyme inhibitors or angiotensin receptor blockers should be preferred, with blood pressure monitoring [61,62].

Tachyarrhythmia may be treated by the use of beta-adrenergic blocking drugs, with protective effect regarding ventricular arrhythmias. Additional therapy can be applied in combination to ERT or chaperone therapy [63,64].

In case of atrial fibrillation, sinus rhythm should be restored, if possible, as brady- or tachyarrhythmia may further reduce the exercise capacity. In these patients, pulmonary vein ablation should be considered. If a restoration is not possible, frequency control should be started and therapy with oral anticoagulants is necessary [10].

In patients with bradycardia beta-adrenergic blocking drugs should be stopped and the implantation of a pacemaker should be considered [18,19]. The insertion of an implantable cardio-defibrillator should be evaluated in patients with life threatening arrhythmias, particularly in patients with end-stage cardiomyopathy [10,47]. 

#### 2.1.2. Future Treatment Options/Investigational Therapies

Results from a phase 1/2 trial showed that pegunigalsidase alfa, a novel pegylated, covalently crosslinked form of alpha-GLA, may represent an advance in ERT, based on its pharmacokinetics and apparent low immunogenicity [65]. Agalsidase alfa and beta exhibit a terminal half-life (T_1/2γ_) of ≤2 h and a maintain measurable plasma level for only <24 h [66,67]. With a T_1/2γ_ of about 80 h, with measurable plasma levels sustained for the entire 2-week dosing interval, pegunigalsidase alfa is providing an active reservoir in the circulation to reach the target tissues [65]. Compared with agalsidase alfa or beta, patients treated with pegunigalsidase alfa developed less treatment induced antidrug antibodies (ADA) [65]. In the BALANCE trial (estimated completion date: May 2022), the BRIDGE trial (enrollment completed December 2019) and the BRIGHT trial (enrollment completed October 2020), the safety and efficacy of pegunigalsidase alpha was investigated [68]. In the BRIDGE trial, an open-label study of the safety and efficacy of pegunigalsidase alfa in patients with FD treated for at least 2 years and on a stable dose (>80%) labelled dose/kg) for at least 6 months with Replagal^®^ (Agalsidase alfa), infusion (1 mg/kg) of the investigational medication has been administered every 2 weeks for 12 months. In the BRIGHT trial, a phase 3, open label, switch over study to assess safety, efficacy and pharmacokinetics of pegunigalsidase alfa 2 mg/kg (Bodyweight) has been administered every 4 weeks for 52 weeks in Fabry disease patients previously treated with ERT (Fabrazyme^®^ (Agalsidase beta) or Replagal^®^ (Agalsidase alfa)) for at least 3 years. For both studies no results have been posted yet.

Substrate reduction therapy (SRT) with Lucerastat is currently under investigation. Lucerastat, or N-butyldeoxygalactonojirimycin (Idorsia Pharmaceutical Ltd., Allschwil, Switzerland), a glucosylceramide synthase (GCS) inhibitor which prevents accumulation of Gb-3 [69] can reduce circulating levels of globotriacylceramide and other sphingolipids [70]. Venglustat (Sanofi Genzyme, Cambridge, MA, USA), another substrate reduction therapy, is currently in a phase 2 study (NCT 02489). With a high volume of distribution Lucerastat may be able to reach tissues that ERT poorly penetrates [69]. In Fabry mice it has been shown that GCS inhibitors can bring reversal of disease phenotypes which are not improved with enzyme replacement therapy [69,71,72]. The data of Welford et al. support the use for Lucerastat in Fabry patients with various genotypes, suggesting that Lucerastat could be an oral therapy suitable for all Fabry patients ignoring the primary mutation [69].

Various approaches to gene therapy are under development. Gene editing occurs ex vivo or in vivo.

With the ex vitro approach, hematopoietic stem cells are harvested from the patient. These cells receive gene editing and are then infused back into the patient for engraftment after myeloablative therapy is conducted. It has been demonstrated that CD34+ positive hematopoietic stem cells could be harvested and modified through recombinant lentivirus (LV)-mediated gene transfer of the GLA gene [73]. In the first gene therapy pilot project for FD, Khan et al. demonstrated efficient LV-mediated gene transfer into enriched Fabry patient CD34+ cells. They reported increased circulating and intracellular GLA activity, without serious safety concerns [74]. All Fabry patients (n = 5) in this pilot trial were responsive to the LV-mediated gene therapy at some level; all patients produced GLA to near normal level within one week, plasma and leukocytes demonstrates GLA activity within or above the reference range, and reductions in plasma and urine globotriaosylceramide (Gb3) and globotriaosylsphingosine (lyso-Gb3) have been demonstrated. Three patients have elected to discontinue enzyme replacement [74]. Persistent elevation of GLA activity in patients has demonstrated early safety of the protocol for this ex vivo approach, as shown also by a press release from AVROBIO [75]. However, before gene therapy can be adopted as therapeutic intervention for FD, a crucial question will be whether the current gene therapy approaches will achieve sufficient GLA activity in different tissues [75].

With the in vivo approach, a vector with gene editing is infused directly into the patient, and then cells within the patient, such as liver cells, directly undergo gene editing to express the missing protein [75]. Pre-clinical data using the liver targeted adeno-associated virus (AAV)-mediated gene transfer (ST920) has shown in alpha-gal A knockout (GLAKO) mouse model in which, after a single injection, GLA is produced by the liver and released into the bloodstream. GLA levels rise in a dose-dependent manner and have achieved levels more than 300 times those of GLA deficient mice [75]. In the first in human treatment with ST920, a recombinant AAV2/6 vector encoding the cDNA for human GAL, the safety and tolerability of ascending doses of ST920 will be elucidated (Sangamo Therapeutics, Brisbane, CA, USA). The estimated primary completion date of this phase 1/2 multicenter study (NCT04046224, ST-920-201) is December 2023 (final data collection date for primary outcome measure) [75]. The trial is currently recruiting participants in the United States. However, gene therapy might have some potential risks: unwanted immune system reactions, targeting false cells, infections induced by the virus and development of a tumor.

Another investigational approach is the administration of GLA mRNA to promote stimulated production of GLA, which is an additional unique form of therapy [76]. It has been shown that mRNA for human GLA encapsulated with lipid nanoparticles could increase GLA levels expressed in cardiac, kidney and liver tissues, resulting in enhanced globotriaosylceramide clearance [76].

## 3. Conclusions

Fabry cardiomyopathy, which leads to reduced life expectancy in untreated patients, can be treated with a causal therapy. However, as myocardial fibrosis is not reversible, the early starting of treatment is crucial for improvement of prognosis. New therapy concepts are under investigation in prospective studies and might help for a more efficient treatment in all stages of the cardiomyopathy.
